# Conessine Interferes with Oxidative Stress-Induced C2C12 Myoblast Cell Death through Inhibition of Autophagic Flux

**DOI:** 10.1371/journal.pone.0157096

**Published:** 2016-06-03

**Authors:** Hyunju Kim, Kang Il Lee, Minsu Jang, Sim Namkoong, Rackhyun Park, Hyunwoo Ju, Inho Choi, Won Keun Oh, Junsoo Park

**Affiliations:** 1 Division of Biological Science and Technology, Yonsei University, Wonju, 26493, Republic of Korea; 2 Korea Bioactive Natural Material Bank, College of Pharmacy, Seoul National University, Seoul, 08826, Republic of Korea; Swedish Neuroscience Institute, UNITED STATES

## Abstract

Conessine, a steroidal alkaloid isolated from Holarrhena floribunda, has anti-malarial activity and interacts with the histamine H3 receptor. However, the cellular effects of conessine are poorly understood. Accordingly, we evaluated the involvement of conessine in the regulation of autophagy. We searched natural compounds that modulate autophagy, and conessine was identified as an inhibitor of autophagic flux. Conessine treatment induced the formation of autophagosomes, and p62, an autophagic adapter, accumulated in the autophagosomes. Reactive oxygen species such as hydrogen peroxide (H_2_O_2_) result in muscle cell death by inducing excessive autophagic flux. Treatment with conessine inhibited H_2_O_2_-induced autophagic flux in C2C12 myoblast cells and also interfered with cell death. Our results indicate that conessine has the potential effect to inhibit muscle cell death by interfering with autophagic flux.

## Introduction

Autophagy (specifically, macroautophagy), a catabolic pathway responsible for degrading protein aggregates and organelles [1], can be induced by extracellular stress (e.g., nutrient starvation, hypoxia, high temperature, and microgravity) [2, 3]. The dynamic process of autophagy, including the conversion of autophagosomes into autolysosomes, is termed autophagic flux, and both the activation and inhibition of autophagic flux result in an increase of autophagosomes in the cytoplasm [4]. The relationship between autophagy and diseases is not clear, however excessive autophagic flux or lack of autophagy can contribute to various diseases such as cancer and neurodegenerative diseases [5–7].

Reactive oxygen species are produced in the mitochondria, and several antioxidant enzymes such as catalase and hydrogen peroxidase are responsible for their removal. Treatment with hydrogen peroxide (H_2_O_2_) can cause oxidative damage and induce autophagy and autophagic cell death under certain conditions [8]. Silencing experiments on autophagy-related genes show that autophagy is involved in ROS-induced cell death [9, 10]. Accordingly, compounds that interfere with excessive autophagic flux may affect muscle viability.

Conessine is a steroidal alkaloid isolated from the bark of Holarrhena floribunda [11]. Recent research has shown that conessine is a potent histamine H3 receptor antagonist by selectively interacting with the histamine H3 receptor, and there have been several attempts to develop new histamine H3 receptor antagonists based on the structure of conessine [12, 13]. Although conessine is also reported to have anti-malarial activity [14, 15], the effect of conessine on cell signaling has not been studied in detail.

We screened autophagy-modulating agents and identified conessine as a negative modulator of autophagy. We found that conessine treatment inhibited autophagic flux and increased the level of p62. Conessine treatment interfered with H_2_O_2_-induced C2C12 cell death. These results suggest that regulation of autophagy by conessine may help prevent muscle cell death.

## Materials and Methods

### Cell culture and cell proliferation assay

HEK293, MCF7 and C2C12 cells were grown in Dulbecco’s Modified Eagle’s medium (DMEM; Welgene, Korea) supplemented with 10% fetal bovine serum (Gibco, Grand Island, NY, USA). A HEK293 stable cell line expressing GFP-LC3 was generated as described previously using a GFP-LC3 plasmid [16]. Transfection of HEK293 and C2C12 cells was performed using lipofectamine (Invitrogen, Carlsbad, CA, USA). Cell proliferation was measured using the [4,5-dimethylthiazol-2-yl]-2,5-diphenyltrazolium bromide (MTT) assay. Briefly, cells were seeded in a 24-well plate and pretreated with conessine for 24 h. Cells were treated with H_2_O_2_ for 24 h and cell proliferation was examined using the MTT assay. Conessine was obtained from the Korea Bioactive Natural Material Bank (KBNMB) and bafilomycin A1 were purchased from Sigma-Aldrich (St. Louis, MO, USA). For cell cycle analysis, transfected cells were washed with phosphate buffered saline (PBS) and fixed with 70% ethanol. After centrifugation, cells were washed and resuspended in PBS containing 0.25 mg/ml propidium iodide (PI) and 10 mg/ml RNase A (Sigma, St. Louis, MO, USA). Cells were analyzed on a FACSCalibur flow cytometer (Beckton-Dickinson, Mountain View, CA, USA). At least 10,000 events per sample were analyzed.

### Western blotting

For protein immunoblot analysis, polypeptides in whole cell lysates were resolved by SDS-PAGE and transferred to nitrocellulose membrane filters. Detection was conducted with a 1:2,000 or 1:5,000 dilution of primary antibody using an enhanced chemiluminescence (ECL) system. Images were acquired using a Chemidoc-it 410 imaging system (UVP, Upland, CA, USA) and LAS4000 system (GE Healthcare, Uppsala, Sweden). The antibody for LC3 was purchased from Novus Biological (Littleton, CO, USA), and the antibody for p62 was from Sigma-Aldrich.

### Immunofluorescence and confocal microscopy

Cells were grown on sterilized glass coverslips. After drug treatment, the cells were fixed with 4% paraformaldehyde. For immunostaining, cells were blocked with 10% bovine serum albumin in PBS and stained with a 1:1,000 dilution of primary antibody in PBS, and then reacted with a 1:1,000 dilution of Alexa 488- or Alexa 568-conjugated secondary antibody (Invitrogen). Finally, the slides were washed three times with PBS, stained with DAPI, and mounted in mounting medium (Vector, Burlingame, CA, USA). Images were captured with a Carl Zeiss LSM710 confocal microscope (Carl Zeiss, Oberkochem, Germany). GFP-mRFP-LC3 (ptfLC3) constructs were purchased from Addgene (Cambridge, MA, USA).

## Results

### Conessine treatment induces autophagosome formation

**Fig 1:**
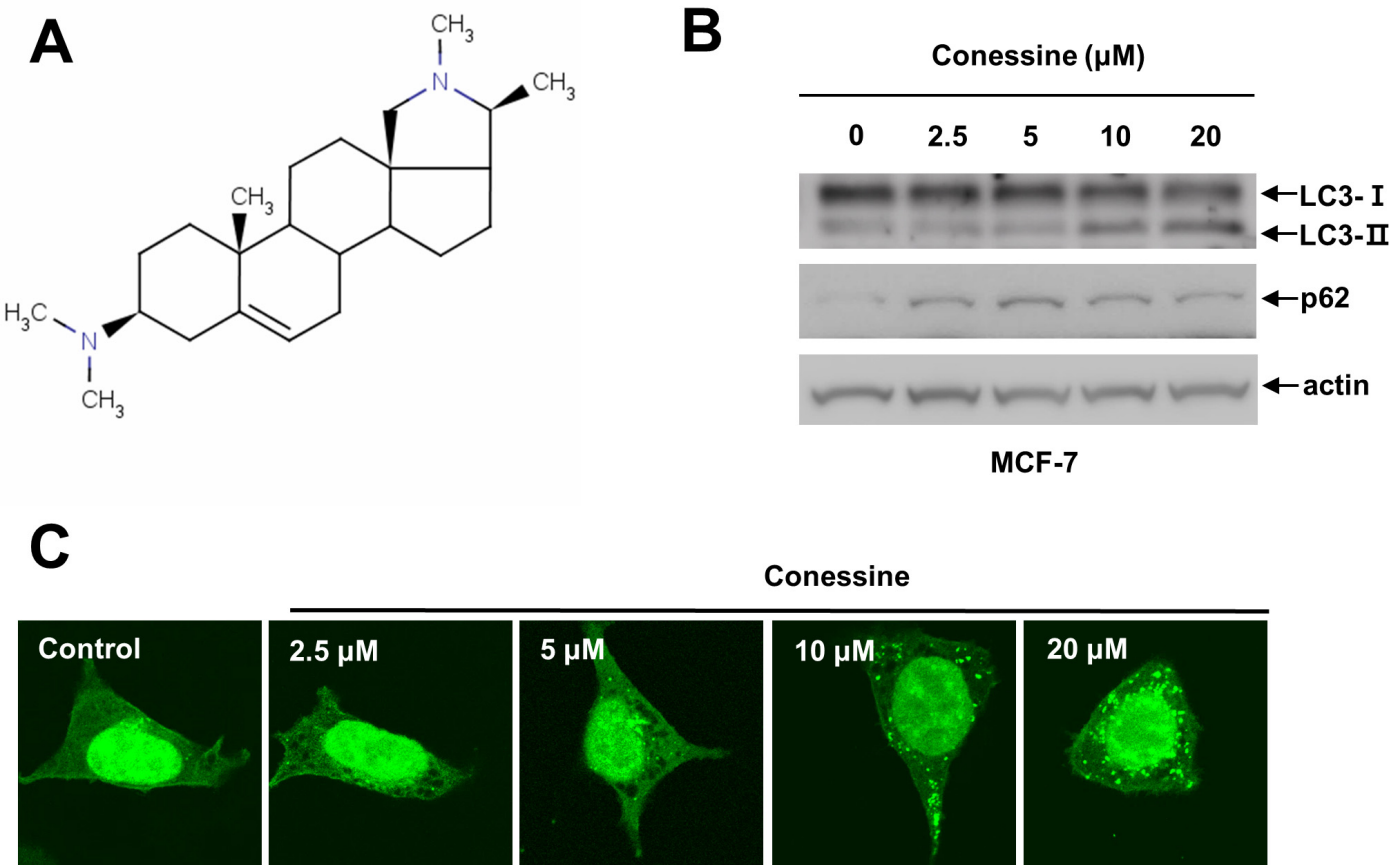
Conessine regulates autophagy. (A) Chemical structure of conessine. (B) Conessine treatment increased the level of LC3-II and p62. MCF-7 cells were incubated with the indicated concentrations of conessine (0, 2.5, 5, 10, and 20 μM) for 24 h, and the cell lysates were subjected to Western blotting with the indicated antibody. (C) Conessine treatment induced autophagosome formation in HEK293 cells. HEK293 cells stably expressing GFP-LC3 were incubated with the indicated concentration of conessine for 24 h, and the cells were analyzed with confocal microscopy.

Recently, the regulation of autophagy has shown promise as a new therapeutic strategy to treat many human diseases such as neurodegenerative diseases and cancers. For this reason, we attempted to identify new compounds that might regulate autophagy. We treated HEK293 cells stably expressing GFP-LC3 with natural single compounds for 24 h and then examined whether cytoplasmic GFP-LC3 punctuates were formed using a fluorescent microscope [17, 18]. This screening strategy identified several compounds, and conessine was identified as a potential autophagy regulator (data not shown). To confirm the screening results, MCF7 cells were treated with various concentrations of conessine (0, 2.5, 5, 10, and 20 μM), and the expression of LC3 protein and p62 protein was evaluated. Conessine treatment increased the LC3-II/LC3-I ratio as well as p62, suggesting that it is involved in autophagy regulation (Fig 1B). We next treated GFP-LC3 cells with various concentrations of conessine and examined the cytoplasmic pattern of GFP-LC3 protein. Cytoplasmic punctuates were evident at a concentration of 10 μM conessine (Fig 1C), providing confirmation that conessine is likely involved in autophagy regulation.

### Conessine treatment inhibits autophagic flux

**Fig 2:**
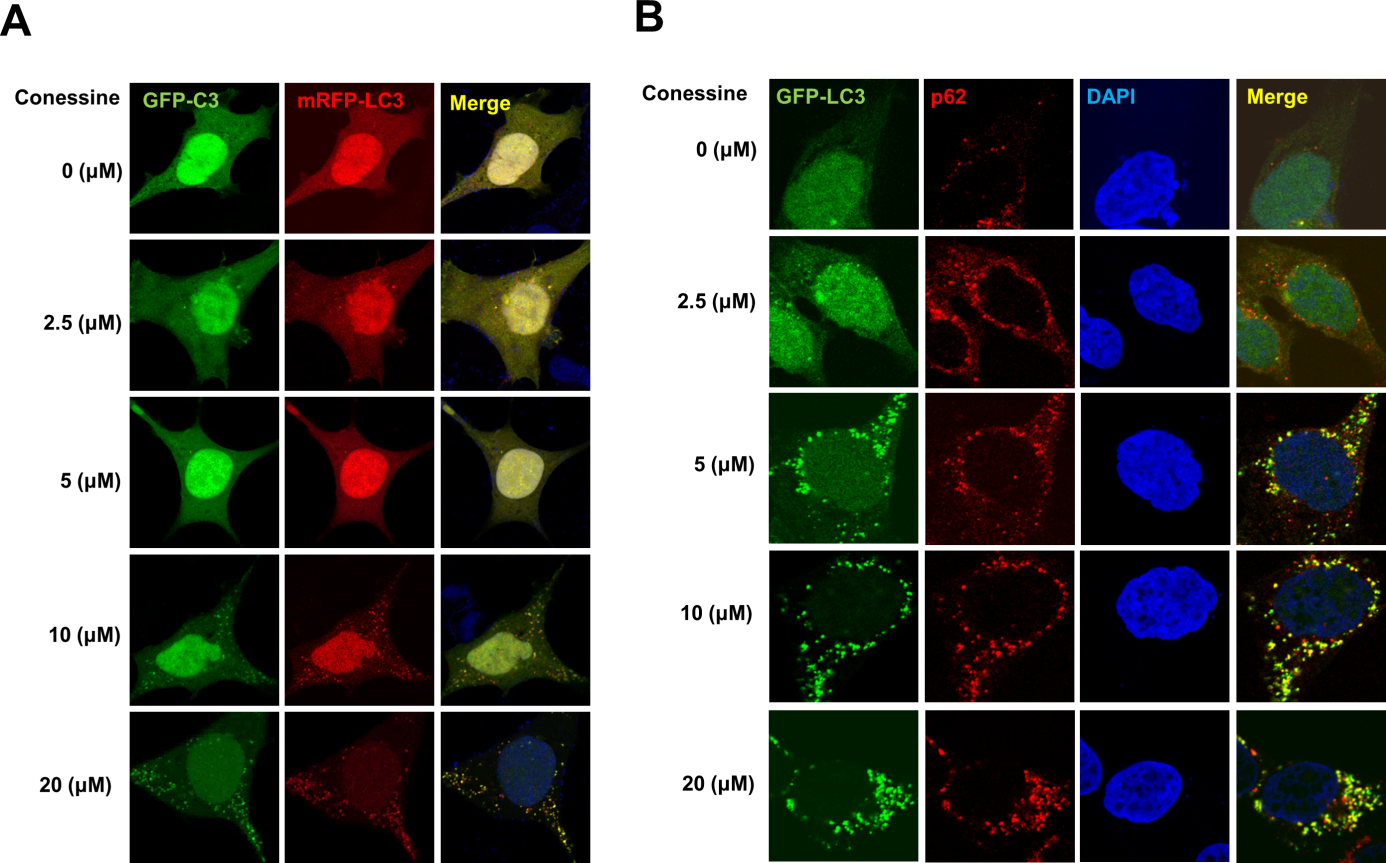
Autolysosomes were not formed by conessine treatment. (A) Autophagosome formation by conessine. HEK293 cells were transfected with a plasmid encoding mRFP-GFP-LC3. After 24h, the transfected cells were incubated with the indicated concentrations of conessine (0, 2.5, 5, 10, and 20 μM) for 24 h. Cells were analyzed with confocal microscopy. (B) p62 accumulated in autophagosomes after conessine treatment. HEK293 cells were transfected with a plasmid encoding GFP-LC3, and the cells were treated with indicated concentrations of conessine, followed by immunostaining with anti-p62 antibody. Cells were analyzed with confocal microscopy.

**Fig 3:**
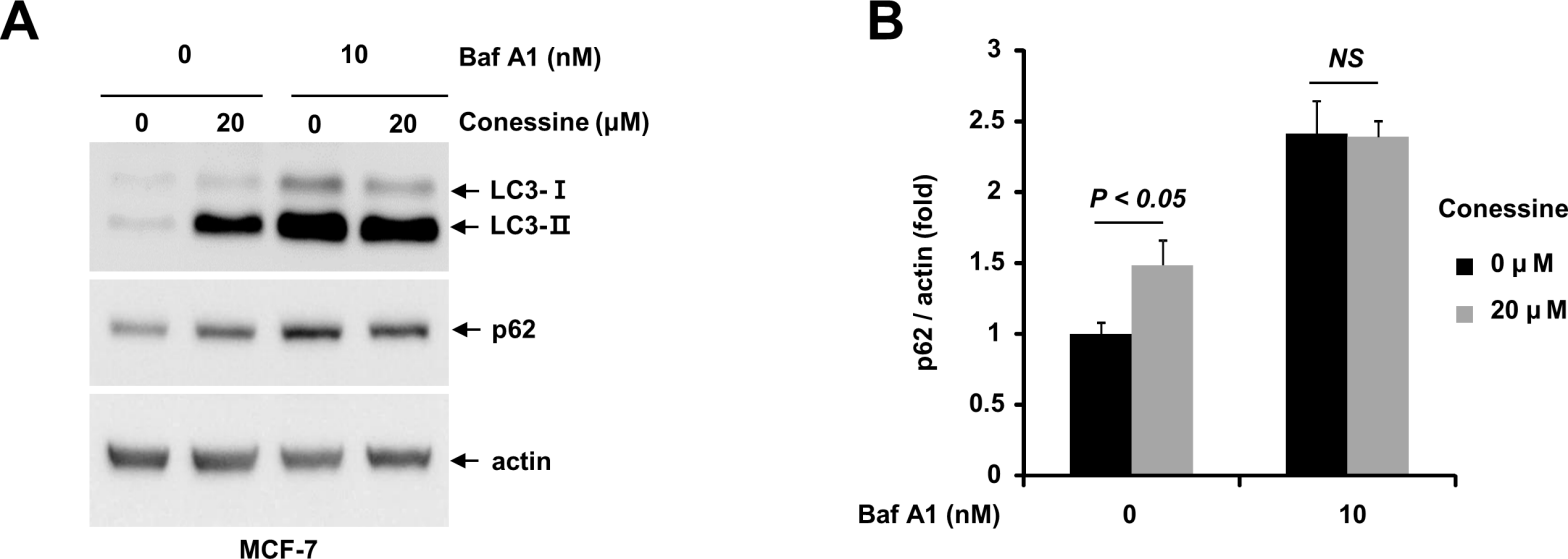
Conessine interferes with autophagic flux. (A) Conessine treatment suppressed with autophagic flux. MCF-7 cells were treated with either mock or conessine (10 μM) in the presence or absence of Bafilomycin A1 (10 nM). (B) The levels of p62 were analyzed. Autophagic flux experiments were performed in triplicate, and the mean and standard deviations are shown in the graph.

Autophagosome formation can be induced either by activation of the autophagy process or by inhibition of autophagic flux [19]. Because we observed increased levels of p62 by Western blot, we hypothesized that autophagic flux was interrupted by conessine treatment. To test our hypothesis, we used GFP-mRFP-LC3 protein to analyze autophagic flux. While both GFP and mRFP are active in autophagosomes, GFP loses its fluorescence in autolysosomes [20]. Treatment with conessine induced the formation of GFP-LC3 punctuates (autophagosomes), however mRFP punctuates (autolysosomes) were rarely detected, suggesting that autophagic flux was interrupted by conessine treatment (Fig 2A). Next, we examined the localization of p62, which accumulated with GFP-LC3 punctuates, indicating that autophagic flux was interrupted (Fig 2B). Finally, we examined autophagic flux using bafilomycin A1, a lysosomal inhibitor. Conessine treatment (10 μM) significantly increased the level of p62, but co-treatment with bafilomycin A1 eliminated the difference in p62 levels between the control and conessine treated samples (Fig 3A and 3B). These results confirm that conessine inhibited autophagic flux (Fig 3A and 3B).

### Conessine attenuates H_2_O_2_-induced myoblast cell death.

**Fig 4:**
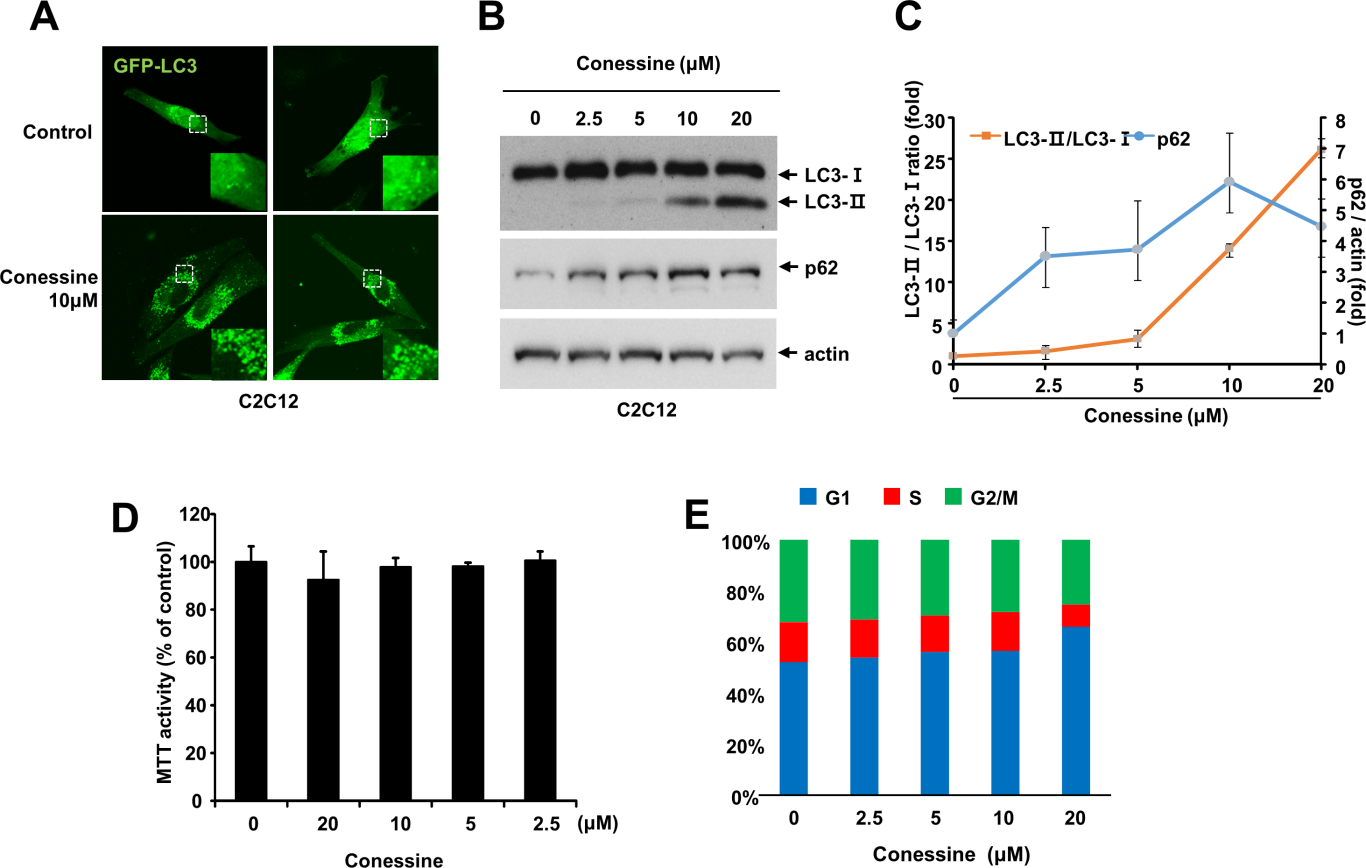
Conessine treatment induces autophagosome formation in C2C12 cells. (A) C2C12 cells were transfected with a plasmid encoding GFP-LC3 and cells were treated with either mock or conessine (10 μM). (B) Conessine treatment increased the level of LC3-II and p62. C2C12 cells were incubated with the indicated concentration of conessine for 24 h, and the cell lysates were subjected to Western blotting with the indicated antibody. (C) The levels of LC3-II/LC3-I and p62 were analyzed. The experiments were performed in triplicate, and the means and standard deviations are shown in the graph. (D) Conessine less than 10 μM did not affect proliferation of C2C12 cells. C2C12 cells were treated with the indicated concentration of conessine, and cell viability were measured by MTT assay (E) Conessine treated cells were analyzed with flow cytometry. The percentage of cells at G1, S and G2/M was measured, and is shown in the graph.

Since conessine interferes with autophagic flux in MCF-7 cells, we wanted to examine whether conessine would also work in muscle cells. For this purpose, we used the C2C12 myoblast cell line. First, we evaluated whether conessine induced the formation of autophagosomes in C2C12 cells. C2C12 cells were transfected with GFP-LC3 plasmids and either mock treated or treated with conessine. Conessine treatment induced the formation of GFP-LC3 punctuates in the cytoplasm (Fig 4A). Next, we examined the expression of autophagic markers LC3 and p62 upon conessine treatment. As expected, conessine treatment increased both the LC3-II/LC-3-I ratio and the level of p62 protein (Fig 4B and 4C). These results indicate conessine inhibited autophagic flux in C2C12 cells.

Oxidative stress can induce muscle cell death by inducing excessive autophagy [9, 10]. Because conessine inhibits autophagic flux, we hypothesized that it interferes with oxidative stress-induced cell death. First, we determined the optimal concentration of conessine treatment. C2C12 cells were incubated with various concentrations of conessine and cell proliferation was examined. Cell proliferation of C2C12 was marginally lower at 20 μM, and cell viability was not affected by conessine up to 10 μM (Fig 4D). Next, we used flow cytometry to analyze the cell cycle after conessine treatment. While there were fewer cells in S and G2/M phase with 20 μM of conessine, the cell cycle was not affected by conessine up to 10 μM (Fig 4E). In addition, the cells in sub G1 phase did not increase after treatment with conessine, indicating that conessine does not induce cell death (Data not shown). These results indicate that conessine up to 10 μM did not affect C2C12 cell viability.

**Fig 5:**
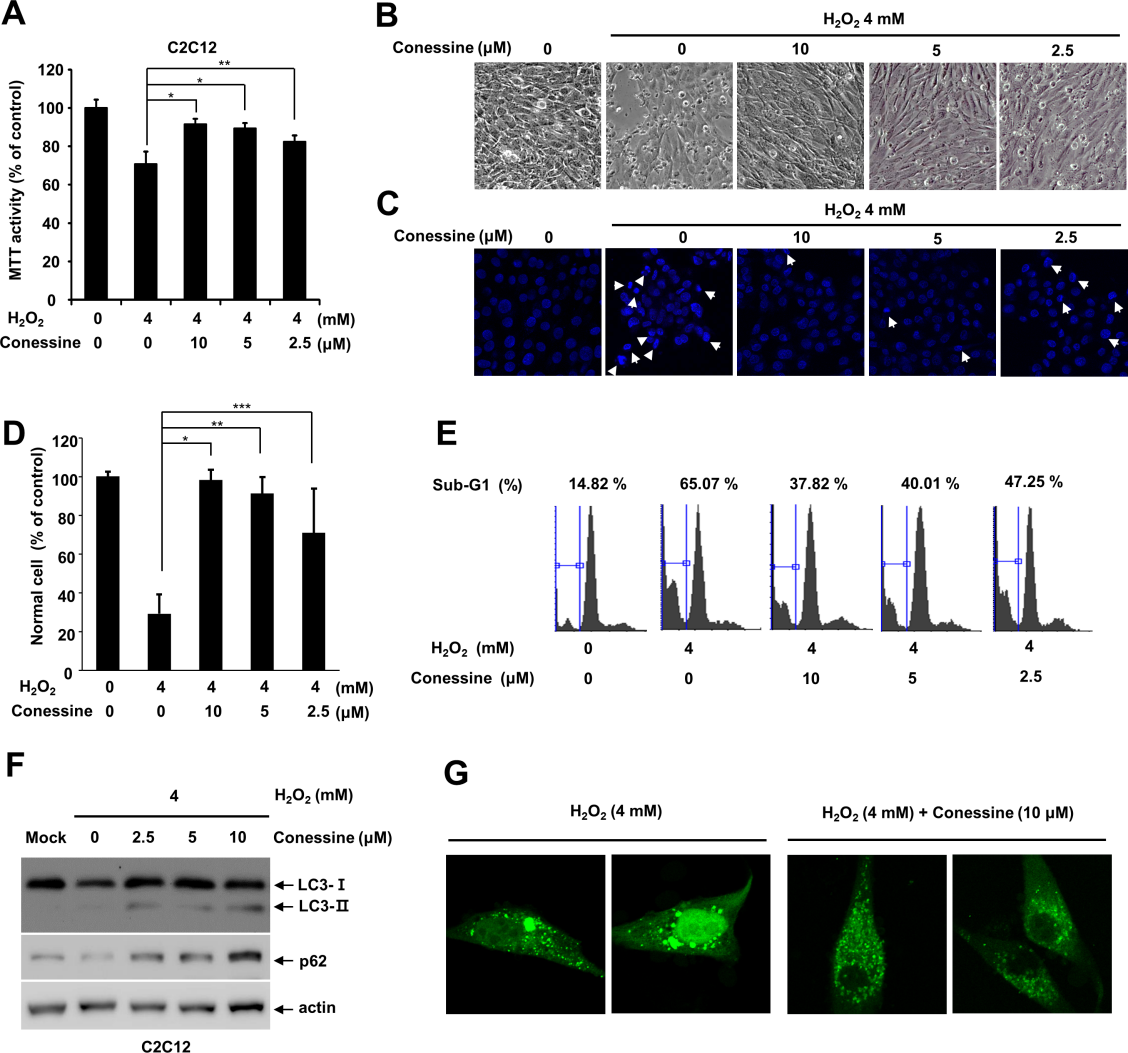
Conessine treatment protects C2C12 myoblast cells from H_2_O_2_-induced cell death. (A) Conessine treatment interferes with H_2_O_2_-induced C2C12 cell death. C2C12 cells were treated with the indicated concentration of conessine, followed by H_2_O_2_ treatment (4 mM) for 24 h. Relative cell viability was measured by MTT assay. Control versus conessine treatment, * *P* < 0.01; ** *P* <0.05 (B) Cellular morphological changes were observed under a phase contrast microscope. (C, D) Apoptotic cells were quantified by counting DAPI-stained nuclei, and apoptotic cells showed nuclei and chromatin condensation. The number of normal cells were shown in graph. At least 160 cells were counted in each samples. Control versus conessine treatment, * *P* < 0.00005; ** *P* <0.0005; *** *P*<0.05. (E) Conessine decreased the sub-G1 cell population, which was induced by H_2_O_2_ treatment. C2C12 cells were treated with conessine and H_2_O_2_, and the cell cycle was analyzed with flow cytometry. (F) Conessine attenuates H_2_O_2_ induced autophagy. C2C12 cells were sequentially treated with conessine and H_2_O_2_, and the cell lysates were subject to Western blot with the indicated antibodies. (G) Conessine alleviates H_2_O_2_-induced excessive autophagy. C2C12 cells were transfected with GFP-LC3 and sequentially treated with conessine and H_2_O_2_. The cells were analyzed by confocal microscopy.

Next, we examined whether conessine interferes with H_2_O_2_-induced myoblast cell death. C2C12 cells were either mock-treated or treated with various concentrations of conessine, followed by treatment with H_2_O_2_. While mock-treated cells showed a significant decline in cell viability, conessine treatment attenuated cell death in a dose-dependent manner (Fig 5A and 5B). In addition, we examined whether conessine interferes with H_2_O_2_-induced cell death in other cell lines. We used mouse fibroblast cell line, NIH3T3, and conessine treatment attenuated H_2_O_2_-induced cell death in NIH3T3 cells (S1 Fig).

To confirm our results, we examined DNA condensation, a feature of apoptosis. C2C12 cells were pretreated with conessine and then incubated with H_2_O_2_ and fixed for DAPI staining. H_2_O_2_ treatment induced up to 70% apoptotic cells with enhanced DAPI staining and conessine treatment decreased the number of apoptotic cells (Fig 5C and 5D). We also examined the cell cycle. While treatment with H_2_O_2_ increased the sub-G1 population up to 71%, conessine treatment decreased the sub-G1 population in a dose-dependent manner (Fig 5E). These results indicate that conessine treatment alleviated H_2_O_2_-induced myoblast cell death.

### Conessine interferes with H_2_O_2_-induced autophagic flux activation

**S**ince conessine attenuated H_2_O_2_-induced cell death, we examined the level of LC3 and p62 after treatment with conessine and H_2_O_2_. H_2_O_2_ treatment decreased p62, suggesting that activation of autophagic flux was induced by H_2_O_2_, but incubation with both conessine and H_2_O_2_ increased the level of p62 to a greater extent (Fig 5F). In addition, the LC3-II/LC3-I ratio also increased after conessine treatment, suggesting that autophagy was modulated by conessine in the presence of H_2_O_2_. These results indicate that conessine interfered with the activation of autophagic flux by H_2_O_2_. Next, we examined GFP-LC3 localization. H_2_O_2_ treatment induced various sizes of GFP-LC3 punctuates in the cytoplasm, and we often observed enlarged autophagosomes, indicating excessive autophagy. However, conessine treatment resulted in uniformly sized autophagosomes and interfered with the formation of enlarged autophagosomes (Fig 5G). These results collectively indicate that conessine interfered with the activation of H_2_O_2_-induced autophagic flux in the myoblast cell line.

## Discussion

Autophagy is involved in various diseases including cancer and neurodegenerative diseases, making autophagy modulators potentially useful for treatment. Rapamycin, an autophagy activator, has been extensively studied for its potential to treat cancer and neurodegenerative diseases [21, 22]. In addition, chloroquine, an autophagic flux inhibitor, has been studied for its potential use for cancer treatment [6]. For this reason, we searched for autophagy modulators using a stable cell line expressing GFP-LC3. With this system, we previously found that amurensin G, an autophagy activator, can attenuate rotenone-induced neuronal cell death, the in vitro Parkinson’s disease model system [17]. Moreover, we showed that reserpine, an anti-hypertensive drug, can contribute to Parkinson’s disease by modulating autophagy [18]. In this report, we demonstrated that conessine has the potential to inhibit autophagic flux. Conessine treatment induces the accumulation of enlarged autophagosomes in the cytoplasm by impairing the fusion of autophagosome with a lysosome (Fig 2). Although we did not identify the molecular targets of conessine to regulate autophagic flux, these findings suggest that conessine potentially interferes with the regulatory proteins which mediate the fusion process of autophagosome with a lysosome, such as Rab proteins, SNARE proteins and v-ATPase [1, 19]. Because muscle cell death and atrophy is related to accelerated autophagic flux, autophagic flux inhibitors like conessine can be useful to prevent muscle cell death. Recently, we also reported that microgravity in space can potentially affect muscle weakening by regulating autophagy, and an autophagic flux inhibitor like conessine would be potentially useful in such a setting [3].

Autophagy is often called a “double-edged sword,” as both excessive autophagy and lack of autophagy can affect cell viability. Likewise, the relationship between autophagy and muscle diseases such as muscle atrophy is not clear yet. Some reports indicate that autophagy is required to prevent muscle atrophy, while others show that autophagy contributes to the induction of muscle atrophy [23, 24]. In this report, we found that conessine, an autophagy inhibitor, interfered with H_2_O_2_-induced myoblast cell death. Because muscle atrophy involves breakdown of aberrant proteins, the inhibition of protein breakdown may delay the onset of muscle atrophy. Further study will be required to determine the long term effect of conessine on muscle atrophy.

## Supporting Information

S1 Fig Conessine treatment protects NIH3T3 fibroblast cells from H2O2-induced cell death. (PDF)
