# Supplementary material for: Conessine Interferes with Oxidative Stress-Induced C2C12 Myoblast Cell Death through Inhibition of Autophagic Flux
Source: PLoS One. 2016 Jun 3;11(6):e0157096. doi: 10.1371/journal.pone.0157096 (PMC4892631; doi:10.1371/journal.pone.0157096)
Supplement: S1 Fig — (PDF) [file pone.0157096.s001.pdf]

Supporting Information

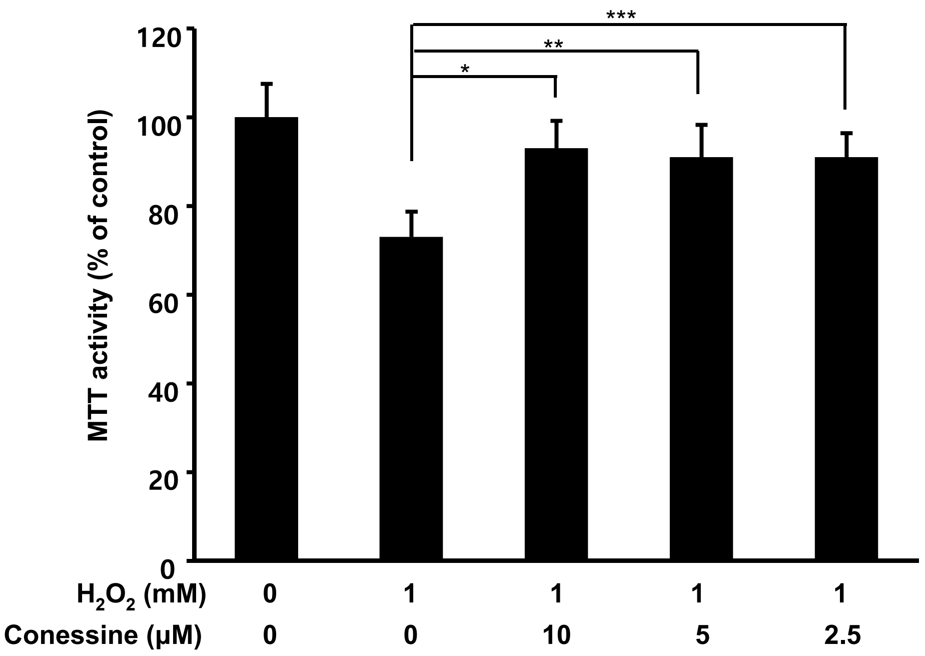

S1-Fig. Conessine treatment protects NIH3T3 fibroblast cells from H_2O_2-induced cell death. NIH3T3 cells were treated with the indicated concentration of conessine, followed by H_2O_2 treatment (1 mM) for 24 h. Relative cell viability was measured by MTT assay. Control versus conessine treatment, * $P < 0.05$; ** $P < 0.001$; *** $P < 0.0001$.
